# LRRC10 regulates mammalian cardiomyocyte cell cycle during heart regeneration

**DOI:** 10.1038/s41536-023-00316-0

**Published:** 2023-07-28

**Authors:** Rebecca J. Salamon, Megan C. McKeon, Jiyoung Bae, Xiaoya Zhang, Wyatt G. Paltzer, Kayla N. Wanless, Alyssa R. Schuett, Dakota J. Nuttall, Stephen A. Nemr, Rupa Sridharan, Youngsook Lee, Timothy J. Kamp, Ahmed I. Mahmoud

**Affiliations:** 1 Department of Cell and Regenerative Biology, University of Wisconsin-Madison School of Medicine and Public Health, Madison, WI 53705 USA; 2 Department of Biomolecular Chemistry, University of Wisconsin-Madison School of Medicine and Public Health, Madison, WI 53705 USA; 3 Department of Nutritional Sciences, Oklahoma State University, Stillwater, OK 74078 USA; 4 Department of Medicine, University of Wisconsin-Madison School of Medicine and Public Health, Madison, WI 53705 USA; 5 Stem Cell and Regenerative Medicine Center, University of Wisconsin-Madison School of Medicine and Public Health, Madison, WI 53705 USA

**Keywords:** Cardiac regeneration, Cytokinesis

## Abstract

Leucine-rich repeat containing 10 (LRRC10) is a cardiomyocyte-specific protein, but its role in cardiac biology is little understood. Recently Lrrc10 was identified as required for endogenous cardiac regeneration in zebrafish; however, whether LRRC10 plays a role in mammalian heart regeneration remains unclear. In this study, we demonstrate that *Lrrc10*^*–/–*^ knockout mice exhibit a loss of the neonatal mouse regenerative response, marked by reduced cardiomyocyte cytokinesis and increased cardiomyocyte binucleation. Interestingly, LRRC10 deletion disrupts the regenerative transcriptional landscape of the regenerating neonatal mouse heart. Remarkably, cardiac overexpression of LRRC10 restores cardiomyocyte cytokinesis, increases cardiomyocyte mononucleation, and the cardiac regenerative capacity of *Lrrc10*^*–/–*^ mice. Our results are consistent with a model in which LRRC10 is required for cardiomyocyte cytokinesis as well as regulation of the transcriptional landscape during mammalian heart regeneration.

The adult human heart shows little regenerative capacity following an ischemic injury, such as a myocardial infarction (MI), in which contractile cardiac muscle is replaced with a fibrotic scar^1^. In contrast, some species of fish and amphibians exhibit the capability of adult heart regeneration in response to injury^2–4^. In addition, neonatal mice and neonatal pigs exhibit a transient potential to undergo heart regeneration after an induced MI^5–7^. New understanding of the mechanisms underlying cardiac regeneration provides opportunities for innovative cardiac regenerative therapies following MI to prevent the progression to heart failure and increased risk of early death^8^.

A recent example of the delicate balance between cardiac regeneration and scar formation following myocardial injury is a study comparing cardiac regeneration in the Pachón cave-dwelling and surface populations of the teleost fish, *Astyanax mexicanus*^9^. Remarkably, this species of fish diverged into cave-dwelling and surface populations 1.5 million years ago and evolved different responses to cardiac injury. The surface fish show robust cardiac regeneration, but little regeneration occurs in the Pachón cave-dwelling fish. Comparison of gene expression profiles following injury revealed that Lrrc10 is more highly expressed in regenerating surface fish. LRRC10 is a cardiomyocyte-specific member of the leucine-rich repeat (LRR) motif family of proteins that mediate protein-protein interactions^10^. Furthermore, *lrrc10* deletion in zebrafish results in loss of cardiac regeneration capacity. In both the Pachón and *lrrc10* knockout zebrafish, there is no difference in the burst in DNA synthesis following injury compared to wild-type controls, but regeneration does not occur, suggesting some impairment of proliferation or cell survival. These results suggest that LRRC10 is essential for cardiac regeneration in fish models; however, the mechanisms remain unclear. In addition, whether LRRC10 is necessary for cardiac regeneration in a mammalian model remains to be understood.

LRRC10 knockout (*Lrrc10*^*–/–*^) mice exhibit mild systolic dysfunction, first detected at embryonic day (E)17.5 without changes in cardiac structure or evidence of fibrosis, and postnatally, the *Lrrc10*^*–/–*^ mice gradually develop a dilated cardiomyopathy but exhibit normal survival^11^. Interestingly, LRRC10 has been recently demonstrated to regulate Ca_v_1.2 channel function and contribute to homeostasis of intracellular Ca^2+^ cycling^12^. In this study, we demonstrate that *Lrrc10* deletion blocks neonatal heart regeneration. Remarkably, *Lrrc10* deletion did not disrupt nuclear division (karyokinesis) during the early stages of M-phase of cardiomyocyte cell cycle, but specifically reduces cardiomyocyte cytoplasmic division (cytokinesis), which results in increased cardiomyocyte binucleation. Transcriptional analysis revealed a unique signature following *Lrrc10* deletion compared to the regenerating control hearts. Importantly, LRRC10 overexpression during neonatal MI rescued the cardiac regenerative capacity of *Lrrc10*^*–/–*^ mice, demonstrating an important role for LRRC10 in regulating cardiomyocyte cytokinesis and cardiac regeneration. Our results reveal a key role for LRRC10 in postnatal cardiomyocyte cell cycle exit and mammalian cardiac regenerative potential.

The role of LRRC10 in mammalian heart regeneration has not been explored. *Lrrc10*^*–/–*^ mice postnatally develop a slowly progressive dilated cardiomyopathy^11^, an indication that LRRC10 is critical for cardiac homeostasis. To determine whether LRRC10 plays a role in mammalian heart regeneration, we evaluated the regenerative capacity of the *Lrrc10*^*–/–*^ mouse heart following an MI in postnatal-day 1 (P1) mice^13^. We utilized the whole-body *Lrrc10* knockout mouse model, *Lrrc10*^*–/–*^, as LRRC10 is a cardiomyocyte-specific protein^14–16^. To determine the effect of *Lrrc10* deletion on myocardial regeneration, we performed Masson’s trichrome staining at 21 days post-MI in wild type (WT) control and *Lrrc10*^*–/–*^ mice. Remarkably, *Lrrc10*^*–/–*^ mice showed increased scar size and incomplete myocardial regeneration in comparison to control hearts (Fig. 1a, Supplementary Fig. 1). Since LRRC10 is a cardiomyocyte-specific protein, this increase in fibrosis is likely due to disruption of the cardiac regenerative response at the cardiomyocyte level rather than an impact on fibroblasts. In addition, we quantified a significant increase in heart weight to body weight ratio in *Lrrc10*^*–/–*^ mice post-MI (Fig. 1b). At 21-days post-sham surgery, no difference in heart weight: body weight was identified between WT and *Lrrc10*^*–/–*^ mice (Fig. 1a, b, Supplementary Fig. 1), indicating there has been no significant remodeling in *Lrrc10*^*–/–*^ hearts by this developmental timepoint. These results demonstrate an evolutionarily conserved role for LRRC10 in regulating cardiac regeneration.

**Fig. 1:**
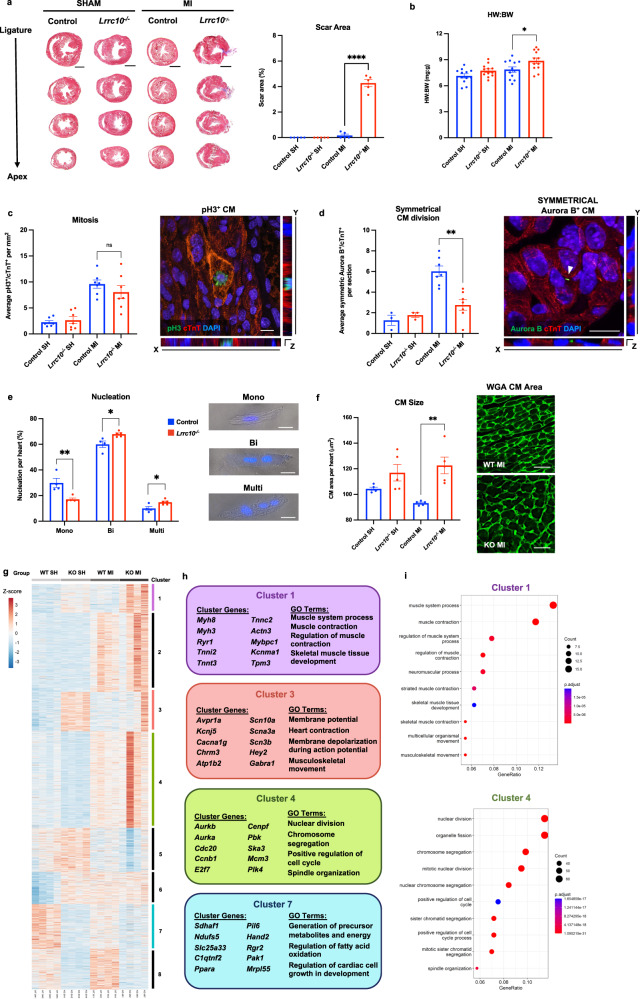
Cardiac regeneration is inhibited in the neonatal *Lrrc10*^*–/–*^ mouse with disruption of the transcriptional landscape following injury. Control and *Lrrc10*^*–/–*^ neonatal mice underwent a sham (SH) or myocardial infarction (MI) surgery at P1 and were analyzed at 7-, 14-, or 21 days post-surgery (DPS). **a** Mason’s trichrome staining (viable tissue in red; scar tissue in blue) at 21DPS (*n* = 5); scale bar =1 mm. **b** Analysis of Heart Weight to Body Weight (BW) ratio (*n* = 12). **c** Analysis of cardiomyocyte mitosis with pH3 and cTnT at 7DPS (*n* = 7); scale bar =10uM. **d** Analysis of cytokinesis by symmetrical localization of Aurora B with cTnT at 7DPS (SH *n* = 3; MI *n* = 7); arrowhead indicates Aurora B localization between cardiomyocytes; scale bar =10uM. **e** Percentage of mononucleated (Mono), binucleated (Bi), and multinucleated (Multi) cardiomyocytes per heart at 14DPS (WT *n* = 4; KO *n* = 5); scale bar =20uM. **f** Quantification of cardiomyocyte size by Wheat Germ Agglutinin (WGA) at 21DPS (*n* = 5); scale bar =25uM. Hearts were collected from WT and *Lrrc10*^*–/–*^ mice at 7 days after sham or MI surgery, processed for bulk-tissue RNA-sequencing (*n* = 4 per group), and the transcriptome of differentially expressed genes (DEGs) was identified. **g** Heatmap generated by k-means clustering of DEGs and divided into 8 clusters; colored scale is related to z-score. **h** Clusters 1, 3, 4, and 7 show a unique transcriptome in *Lrrc10*^*–/–*^ hearts. **i** Gene Ontology (GO) analysis highlights LRRC10 dysregulated pathways related to muscle contraction (Cluster 1) and cell metabolism (Cluster 4). *P $\le$ 0.05, **P $\le$ 0.01, ****P $\le$ 0.0001. Error bars presented as S.E.M.

Heart regeneration is mediated by the proliferation of the pre-existing cardiomyocytes^5^. Here, we analyze two stages of M-phase during cardiomyocyte cell cycle. We use phospho-Histone3 (pH3) as a marker for early G2-M phase to measure karyokinesis. In addition, we use Aurora B as a marker of cytokinesis, which is symmetrically localized at the cleavage furrow to measure cytoplasmic division. During mammalian heart regeneration, cardiomyocyte proliferation peaks around 7 days post-MI^5,17^. To establish the impact of Lrrc10 deletion on cardiomyocyte proliferation and division, we performed immunostaining for the early M-phase marker pH3 and the cardiomyocyte marker cardiac troponin T (cTnT) at 7 days following MI at P1. We found no significant difference in levels of pH3 positive cardiomyocytes between WT control and *Lrrc10*^*–/–*^ mice (Fig. 1c, Supplementary Fig. 2A, B). Our results are similar to the prior cavefish and zebrafish study, which showed comparable levels of induced DNA synthesis in both controls and *lrrc10*^*–/–*^ zebrafish at the same timepoint^9^. However, mammalian cardiomyocytes are prone to multinucleation and increased ploidy following DNA synthesis, a phenomenon that contributes to the loss of regenerative capacity in mice^18^. Localization of the cytokinesis marker Aurora B can distinguish between events of complete cytokinesis and binucleation in cardiomyocytes. Symmetrical localization of Aurora B between two nuclei is indicative of cytokinesis, whereas asymmetrical localization of Aurora B can indicate binucleation^19,20^. To determine whether LRRC10 regulates later stages of cardiomyocyte cell cycle, we measured levels of the symmetric and asymmetric Aurora B localization in cardiomyocytes at 7 days post-MI. Remarkably, we detected a significant decrease in the number of symmetrically localized Aurora B cardiomyocytes in *Lrrc10*^*–/–*^ mice compared to control mice following MI (Fig. 1d, Supplementary Fig. 2C, D). There was no significant difference in the number of cardiomyocytes with asymmetric Aurora B localization between WT and *Lrrc10*^*–/–*^ mice (Supplementary Fig. 2E). Interestingly, at 7 days-post sham surgery, WT and *Lrrc10*^*–/–*^ mice had comparable levels of cardiomyocytes positive for pH3 (Fig. 1c), as well as symmetrical and asymmetrical Aurora B localization (Fig. 1d, Supplementary Fig. 2E), indicating that the impact of Lrrc10 on cardiomyocyte cytokinesis is an injury-specific response that is only evident during regeneration. These results suggest that LRRC10 specifically regulates cardiomyocyte cytokinesis during cardiomyocyte proliferation and heart regeneration.

To further establish the impact of *Lrrc10* deletion on cardiomyocyte nucleation, we isolated cardiomyocytes from control and *Lrrc10*^*–/–*^ mice at 14-days post-injury and quantified nucleation with the DNA dye Hoechst. We measured a significant decrease in mononucleated cardiomyocytes, as well as a significant increase in binucleated and multinucleated cardiomyocytes in *Lrrc10*^*–/–*^ mice post-MI compared to control mice (Fig. 1e). No significant difference in nucleation levels were identified between sham control and *Lrrc10*^*–/–*^ mice (Supplementary Fig. 2F), further confirming that Lrrc10 deletion does not impact cardiomyocyte cell cycle and nucleation in the absence of injury. Furthermore, we quantified a significant increase in cardiomyocyte size in *Lrrc10*^*–/–*^ mice compared to controls post-MI by Wheat Germ Agglutinin (WGA) staining, without significant changes in cardiomyocyte area post-sham surgery (Fig. 1f).

Collectively, our results demonstrate that *Lrrc10* deletion specifically impedes cardiomyocyte cytokinesis but not karyokinesis following injury, which results in increased cardiomyocyte nucleation and blockade of the neonatal cardiac regenerative response. Thus, LRRC10 plays a conserved role in heart regeneration across permissive species in controlling the late stages of cardiomyocyte proliferation prior to completion of cell division.

Our results demonstrate that LRRC10 regulates cardiomyocyte cytokinesis and mammalian heart regeneration post-MI. The endogenous heart regenerative response of the neonatal mouse heart is regulated by a unique transcriptional landscape^21–23^. To identify how LRRC10 mediates cardiomyocyte cytokinesis and cardiac regeneration at the transcriptional level, we analyzed the global transcriptome in control and *Lrrc10*^*–/–*^ hearts. We performed bulk RNA sequencing on the ventricles from WT control and *Lrrc10*^*–/–*^ mice that underwent a sham or MI surgery at P1 (Fig. 1g–i). Hearts were collected at 7 days post-injury from equal numbers of male and female mice. Analysis of the transcriptomic landscape by Principal Component Analysis (PCA) and Pearson correlation demonstrates distinct clustering between control and *Lrrc10*^*–/–*^ sham and MI groups (Supplementary Fig. 3A, B), indicative of a unique transcriptional signature in *Lrrc10*^*–/–*^ mice following injury.

To further dissect the transcriptional signature related to the regenerative defects following *Lrrc10* deletion, we defined the differentially expressed genes (DEGs) and performed K-means clustering and Gene Ontology (GO) analysis of the DEGs. We identified 8 total clusters with distinct transcriptomic patterns (Fig. 1g). Most interestingly, Cluster 1 shows a transcriptomic signature only upregulated in the *Lrrc10*^*–/–*^ mice post-MI (Fig. 1g, Cluster 1). The clustered genes encode for myofilament proteins, such as myosins (*Myh1, Myh3, Myh8*), troponins (*Tnnt3, Tnnc2, Tnni2*), and actin (*Actn3*) proteins (Fig. 1h). Furthermore, GO analysis identified cell processes related to regulation of muscle contraction and development (Fig. 1g–i). This increased expression of myofilament proteins maybe a compensatory effect, as recent evidence demonstrates that LRRC10 modulates Ca_v_1.2 calcium channels and cardiomyocyte contraction^12^, while LRRC10 deletion results in development of dilated cardiomyopathy later in life^11^.

Cluster 3 shows a distinct transcriptome in both sham and MI *Lrrc10*^*–/–*^ mice compared to controls, which includes genes encoding for major ion channels, such as a voltage-gated T-type Ca^2+^ channel (*Cacna1g*), sodium channels (*Scna10a, Scn3b*), and a potassium channel (*Kcnaj5*) (Fig. 1g, h, Cluster 3). GO analysis related these channel-encoding genes to regulation of cardiac contraction, membrane potential, and depolarization (Fig. 1h). Changes in ion channel expression can be linked to a variety of cardiac pathologies. For example, mutations in sodium channels can lead to ventricular arrythmias in humans, and expression of T-type Ca^2+^ channel genes are associated with cardiac hypertrophy^24,25^.

The transcriptome in Cluster 4 reveals a specific gene expression in *Lrrc10*^*–/–*^ samples post-MI compared to controls (Fig. 1g, Cluster 4), suggesting this cluster contained a distinct transcriptomic profile linked to the injury response of *Lrrc10*^*–/–*^ hearts. Further analysis identified genes associated with cell cycle such as *Cdc20, Ccnb1*, and the related GO pathways involved in cell cycle regulation (Fig. 1h, i). Interestingly, one of the downregulated genes in the cluster, *Aurkb*, encodes for the cytokinesis regulator Aurora B kinase, supporting our earlier evidence that *Lrrc10*^*–/–*^ inhibits the completion of cardiomyocyte division (Fig. 1d).

Lastly, we investigated the transcriptome in Cluster 7, as the overall gene expression was downregulated in *Lrrc10*^*–/–*^ sham and MI mice compared to controls. Genes and related GO terms highlight an association to cell metabolism, such as fatty acid oxidation and mitochondrial metabolism (*Sdhaf1, Ndufs5, Slc25a33*) (Fig. 1g, h, Cluster 7). The cardiac metabolic state plays an important role in mediating endogenous heart regeneration, where altering the balance between glucose and fatty acid oxidation metabolism can promote or inhibit regeneration, respectively^26^. This transcriptional signature demonstrates that *Lrrc10* deletion may drive metabolic dysregulation and result in the impaired regenerative response to injury.

Our analysis highlights important roles for LRRC10 in transcriptional regulation of key cell processes related to muscle contraction, ion-channel function, cell cycle activity, and metabolism. Together, this demonstrates that *Lrrc10*^*–/–*^ mice have a unique transcriptional signature underpinning the loss of the cardiac regenerative capacity.

Our results demonstrate that loss of LRRC10 blocks neonatal heart regeneration. However, whether lack of LRRC10 is primarily responsible for the defect in neonatal heart regeneration rather than secondary effects on the heart remains unclear. Thus, we wanted to determine whether restoration of LRRC10 protein levels can rescue the blockade of the cardiac regenerative response in post-natal *Lrrc10*^*–/–*^ hearts. (Fig. 2a). To address this question, we injected WT and *Lrrc10*^*–/–*^ mice at P0 with a single dose of AAV9-cTnT-GFP control vector or AAV9-cTnT-LRRC10 rescue vector, for cardiac-specific overexpression of LRRC10, followed by an MI surgery at P1 (Fig. 2a). This dosing strategy was sufficient to target the heart, as evident by GFP expression throughout the heart by 7 days post-injection (Fig. 2b, Supplementary Fig. 4). We first investigated if the overexpression of LRRC10 in *Lrrc10*^*–/–*^ hearts was sufficient to rescue the defect of symmetrical Aurora B localization in cardiomyocytes. Interestingly, at 7 days post-MI we measured a significant increase in cytokinesis in *Lrrc10*^*–/–*^ mice treated with AAV9-cTnT-LRRC10 rescue vector compared to the AAV9-cTnT-GFP control vector (Fig. 2c). There was no significant difference in asymmetrical Aurora B localization between the same groups (Supplementary Fig. 5), in line with our earlier results demonstrating comparable levels of asymmetrical Aurora B between WT and *Lrrc10*^*–/–*^ hearts post-MI (Fig. 1d).

**Fig. 2:**
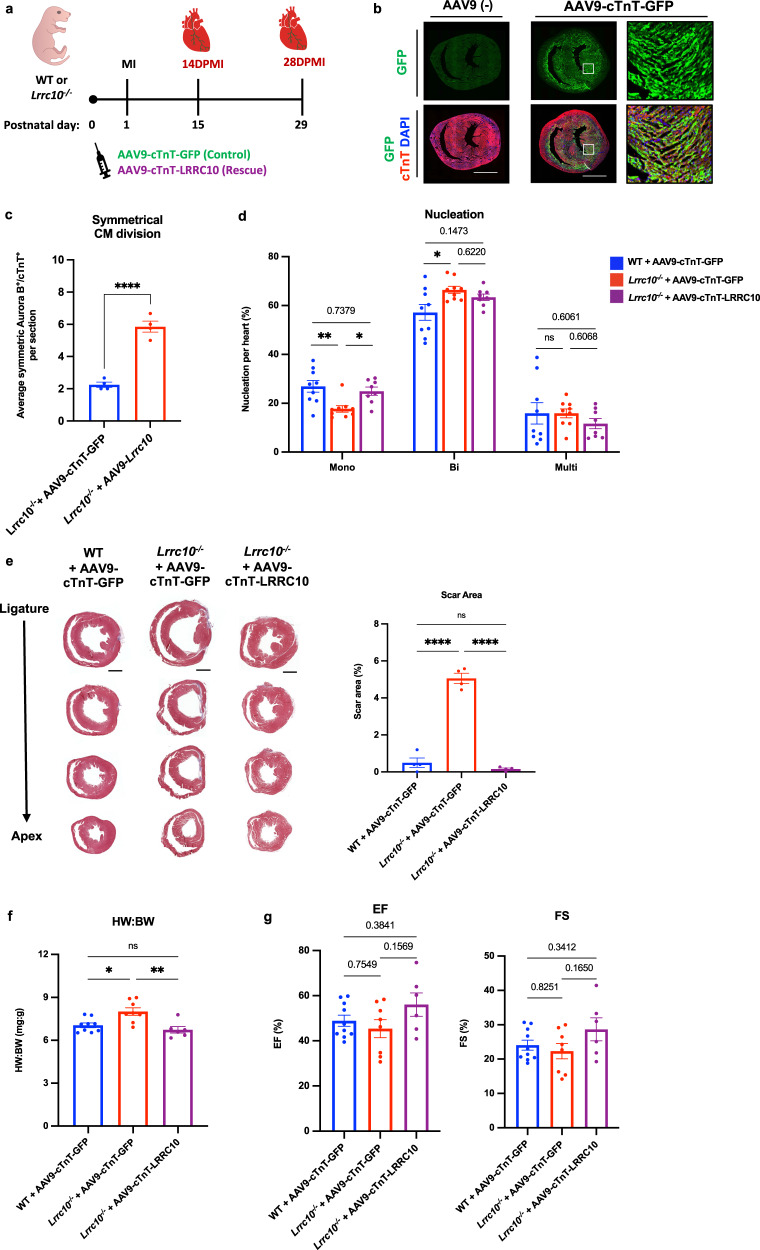
LRRC10 overexpression rescues heart regeneration in *Lrrc10*^*–/–*^ mice. **a** Control and *Lrrc10*^*–/–*^ neonatal mice were treated with a single-dose of AAV9-cTnT-GFP control or AAV9-cTnT-LRRC10 rescue viral vector before undergoing sham or MI surgery at P1. **b** Efficient transfection shown by GFP expression in WT P8 mice treated with or without AAV9-cTnT-GFP at P0 (*n* = 3). Hearts were analyzed for regeneration hallmarks at 7-, 14- and 28-days post-MI (DPMI). **c** Cytokinesis measured by symmetrical Aurora B within cardiomyocytes, marked by cTnT at 7DPMI (*n* = 4). **d** Nucleation percentages of mononucleated (Mono), binucleated (Bi), and multinucleated (Multi) cardiomyocytes (WT and KO controls *n* = 9; KO rescue *n* = 8) at 14DPMI. **e** Masson’s trichrome analysis at 28DPMI (*n* = 4); scale bar =1 mm. **f** Heart weight to body weight (BW) ratios at 28DPMI (WT control *n* = 9; KO control *n* = 8; KO rescue *n* = 6). **g** Echocardiography of ejection fraction (EF) and fractional shortening (FS) at 28DPMI (WT control *n* = 9; KO control *n* = 8; KO rescue *n* = 6). *P $\le$ 0.05, **P $\le$ 0.01, ****P $\le$ 0.0001. Error bars presented as S.E.M.

We further investigated the effect of LRRC10 overexpression on cardiomyocyte cytokinesis by quantifying cardiomyocyte nucleation as a readout of cardiomyocyte division across control and rescue groups. At 14 days post-MI, LRRC10 rescue hearts restored the increase in mononucleated cardiomyocytes compared to the LRRC10 KO controls (Fig. 2d). Furthermore, the number of binucleated and multinucleated cardiomyocytes in LRRC10 rescue hearts were restored to the levels of WT control hearts, whereas LRRC10 KO controls show a significant elevation in binucleated cardiomyocytes (Fig. 2d). This demonstrates that overexpression of LRRC10 in *Lrrc10*^*–/–*^ mice restores the increase in mononucleated cardiomyocytes post-MI.

To determine whether LRRC10 overexpression restores myocardial regeneration and scar size reduction in *Lrrc10*^*–/–*^ mice, we performed trichrome staining at 28 days post-MI. LRRC10 KO control mice show incomplete regeneration, persistent scar tissue, and thinning of the left ventricle, as expected (Fig. 2e, Supplementary Fig. 6). Strikingly, the LRRC10 rescue mice show structural regeneration, with little to no scarring present and increased wall thickness in the left ventricle, similar to WT controls. In addition, LRRC10 rescue mice also demonstrate a reduction of heart weight to body weight ratio, restoring heart size to similar levels as WT controls (Fig. 2f). To identify if this repair translated to improved cardiac function, we used echocardiography to measure ejection fraction (EF) and fractional shortening. Hearts of LRRC10 rescue mice showed trending improvements in EF and FS compared to LRRC10 KO control hearts (Fig. 2g). The restoration of myocardial structure and scar resolution demonstrate that LRRC10 overexpression can promote heart regeneration in *Lrrc10*^*–/–*^ hearts (Fig. 2e–g). Together, these results demonstrate that the inhibition of regeneration in *Lrrc10*^*–/–*^ mice is driven by reduced levels of LRRC10 and that LRRC10 overexpression postnatally is sufficient to restore myocardial regeneration following neonatal MI.

Heart failure with reduced ejection fraction following myocardial infarction remains a major health and economic burden given the inability of the adult mammalian heart to regenerate following injury. Defining the mechanisms that control endogenous heart regeneration can identify new therapeutic approaches to promote adult human heart regeneration. Our results demonstrate an evolutionarily conserved role for LRRC10 in heart regeneration, from the *Astyanax mexicanus* surface fish and zebrafish to the neonatal mouse. More importantly, we demonstrate that LRRC10 regulates later stages of cardiomyocyte cell cycle activity that impacts cardiomyocyte division and nucleation during neonatal mouse heart regeneration. Loss of LRRC10 results in transcriptional dysregulation of muscle, ion channel, cell cycle, and metabolic genes, which may play a role in impeding heart regeneration. Remarkably, restoration of LRRC10 levels in *Lrrc10*^*–/–*^ hearts is sufficient to rescue the endogenous regenerative response. Our results suggest a unique role for LRRC10 in regulating cardiomyocyte cytokinesis but not karyokinesis, demonstrating a stage-specific regulation of cardiomyocyte cell cycle.

One limitation of this study is that quantification of Aurora B localization from in vivo heart sections, as well as cardiomyocyte nucleation from dissociated cardiomyocytes, cannot definitively prove cytokinesis. Although cardiomyocyte cytokinesis can be directly visualized via live imaging in vitro^27,28^; the impact of LRRC10 on cardiomyocyte Aurora B labeling and nucleation is only evident in response to injury and subsequent regeneration. Thus, live imaging of cultured cardiomyocytes cannot be utilized to directly visualize the effect of LRRC10 on cardiomyocyte cytokinesis in response to neonatal heart injury.

Owing to the identified role of LRRC10 in regulating L-type Ca^2+^ channels in adult cardiomyocyte, future studies are warranted to further define the impact of calcium handling on cardiomyocyte proliferation and heart regeneration. Furthermore, the impact of LRRC10 deletion on cardiac metabolism needs to be further investigated, which might reveal a link between calcium homeostasis and cardiomyocyte cell cycle. In addition, whether overexpression of LRRC10 in the adult heart can promote adult cardiomyocyte cell cycle re-entry and regeneration remains to be determined. This study provides an important new target to modulate cardiomyocyte cell cycle activity and heart regeneration.

## Methods

### Animals

Wild type C57BL/6 J (Stock #000664) mice were obtained from Jackson Laboratories. *Lrrc10*^*–/–*^ mice were generated in C57BL/6 background and genotyped as described previously^11,29^. All animal experimental procedures were approved by the Institutional Animal Care and Use Committee of the University of Wisconsin-Madison. All experiments were performed on age and sex matched mice, and RNA-seq analysis was performed with an equal ratio of male to female mice.

### Neonatal myocardial infarction

Neonatal mice underwent myocardial infarction (MI) surgery at postnatal day 1, as previously described^13^. Briefly, neonates were anesthetized by hypothermia on ice. A blunt dissection was performed in the fourth intercostal space. The heart was gently guided to rest on the chest cavity and the LAD was located. Using a C-1 tapered needle with a 6-0 Prolene suture (Ethicon Inc., Bridgewater, NJ), the LAD was ligated and blanching at the apex was visualized. The heart was guided back into the chest, the ribs were sutured closed, and skin was joined using adhesive glue (3 M). The mice recovered on a warmed heating pad until mobile. The sham operation consisted of hypothermic anesthesia, blunt dissection in the fourth intercostal space, and closing of the chest cavity, without heart exposure or LAD ligation.

### Histology

For paraffin embedding, hearts were fixed in 4% paraformaldehyde (PFA) in PBS at 4 °C overnight. Samples were embedded in a paraffin block and sectioned below the ligation at 5um thickness. Masson’s trichrome stain was run according to the manufacturer’s protocol (Newcomer Supply, Middleton, WI). Scar area quantified in ImageJ and averaged across 3 sections per heart.

For cryosections, hearts were fixed in 4% PFA in PBS at RT for 1 hr. Tissues were soaked in 30% sucrose overnight before being submerged into cryomold with OTC and frozen at −80 °C. Hearts were sectioned at 8um thickness.

### Immunostaining

Paraffin sections underwent deparaffinization and rehydration by sequential 3 min incubations in xylene and ethanol (100%, 90%, 70%) solutions. Samples were placed into IHC antigen retrieval solution (Invitrogen, Carlsbad, CA) and microwaved for 10 min. Sections were blocked in 10% blocking serum (matching secondary) and incubated in primary antibodies overnight incubation at 4 °C. Primary antibodies were used against phospho-Histone3 Ser10 (Millipore, catalog # 06-570) at [1:200] dilution, Aurora B (Sigma, catalog # A5102) at [1:100] dilution, and Cardiac Troponin T (cTnt Abcam, catalog # AB8295) at [1:200] dilution. Sections were washed with PBS and incubated with secondary antibodies (Invitrogen) at [1:400] dilution with DAPI for 1 hour at room temperature. Slides were mounted in antifade mounting medium and stored at 4 °C. Representative images were taken on a Nikon A1RS HD confocal microscope.

Cardiomyocyte cross-sectional area was measured by Wheat Germ Agglutinin (WGA) staining. Paraffin sections were processed as described above, incubating with WGA-488 conjugated antibody (Thermo Fisher, catalog # W11261) at [1:50] dilution and cTnT. Cardiomyocyte area was quantified by measuring cross-sectional area of cardiomyocytes dual-positive for WGA+ and cTnT in Image J, measuring approximately 200 cardiomyocytes across 4-6 replicate sections.

For GFP staining in AAV9-injected mice, cryosections were placed into a humidifying chamber and incubated with 10% blocking buffer, diluted in PBS with 0.2% Triton X-100 (PBST) at RT for 1 hr. Slides were incubated with primary GFP-488 conjugated antibody (Thermo Fisher, catalog #A21331) and cTnT diluted in PBST with 5% blocking buffer at 4 °C overnight. Sections were washed, mounted, and stored at −20 °C. Representative images were taken on a Nikon A1RS HD confocal microscope. For whole-mount staining, the whole heart was harvested, washed in PBS and immediately imaged for endogenous GFP expression. Representative images were acquired on a Nikon Upright FN1 confocal microscope.

### AAV9 injection

Adenovirus vector constructs, AAV9-cTnT-EGFP-WPRE (catalog # VB5428) and AAV9-cTnT-mLRRC10-WPRE were designed and produced by Vector Biolabs (Malvern, PA). Mice were injected subcutaneously at P0 with AAV9 constructs at a viral titer of 5 × 10^13^ vg/kg BW (diluted in saline to a total volume of 10ul). MI was performed at P1, as described above, and hearts were collected at 7 days-post MI for cytokinesis analysis, 14 days post-MI for nucleation analysis, and 21 days post-MI for histological analysis.

### Cardiomyocyte isolation and nucleation

Mice hearts were harvested at 14 days post-surgery and fixed in 4% PFA in PBS at RT for 2 h. Hearts were washed for three, 15 min incubations in PBS. Hearts were mined into 1 mm pieces and transferred into Eppendorf tubes containing collagenase solution with collagenase D (2.4 mg/ml, Cat #11088866001) and collagenase B (1.8 mg/ml, Cat #: #110088807007) diluted in Hank’s Balanced Salt Solution (Santa Cruz, #sc-391061A) and incubated on a rocker at 37 °C overnight. Collagenase solution was replaced every two days by centrifugation at 500 g for 1 min at RT and removing supernatant. After cells were dissociated, cell pellets were collected by centrifugation at 500 g for 2 min at RT, resuspended in PBS, and passed through a 100 µm cell filter to purify cardiomyocyte populations and remove clumps. Cells were stored at 4 °C until ready for staining.

Nucleation staining and quantification was performed on isolated cardiomyocytes. Cells were mixed gently to resuspend and 200ul of cardiomyocytes were transferred to a 1.5 ml Eppendorf tube with 300ul of PBST (PBS with 0.2% Triton X-100). DAPI was added to solution and incubated for 10 min at RT. After, 1 ml of 10% blocking buffer was added to reduce cell clumping. Cells were collected by centrifugation at 800 g for 2 min and supernatant was discarded, leaving approximately 100ul of solution. For nucleation quantification, around 300-500 cardiomyocytes were aliquoted onto a coverslip and sealed with nail polish. Approximately 1000 intact cardiomyocytes were counted per heart, with 4-9 replicate hearts per sample group. Nucleation distribution was presented as percent nucleation of total cardiomyocytes per sample.

### RNA sequencing and analysis

Sex-matched heart ventricles were collected at 7 days post-surgery (Sham or MI) and immediately homogenized in Trizol (Invitrogen) according to the manufacturer’s protocol. Mouse tissue samples suspended in TRIzol were submitted to the University of Wisconsin Biotechnology Center (UWBC) Gene Expression Center (*Research Resource Identifier - RRID:SCR_017757)* for RNA extraction. Total RNA was purified following the recommendations of the Qiagen RNeasy Mini (Qiagen, Hilden, Germany) procedure, which included on-column DNase treatment. RNA quality and integrity (RINe > 8.4) were verified on a NanoDrop One Spectrophotometer (Thermo Fisher Scientific, Waltham, MA, USA) and Agilent 4200Tapestation (Santa Clara, CA, USA), respectively.

Total RNA was used as input material and libraries were prepared by following the SMARTer Stranded Total RNA Sample Prep Kit – HI Mammalian user manual (Takara Bio USA, Mountain View, CA, USA). In brief, 900 ng total RNA were hybridized to RiboGone™ oligos for depletion of rRNA sequences by RNase H-mediated digestion followed by SPRI bead cleanup. Reduced rRNA templates were fragmented at 94 °C for 3 min prior to first-strand synthesis. Takara adaptors and indexes were added to single-stranded cDNA via 12 cycles of PCR. Quality and quantity of the finished libraries were assessed on the Agilent 4200 Tapestation (Agilent, Santa Clara, CA, USA) and Qubit Fluorometer (Invitrogen, Carlsbad, CA, USA), respectively. Paired end 150 bp sequencing was performed by Illumina Sequencing by UWBC DNA Sequencing Facility *(RRID:SCR_017759)* on an Illumina NovaSeq6000, with libraries multiplexed for an approximate 50 million reads per library. Sequencing was done using standard 300 cycle TruSeq v1.5 SBS kits and SCS 2.8 software. Images were analyzed using the standard Illumina Pipeline, version 1.8.

Bioinformatic analysis of transcriptomic data adhere to recommended ENCODE guidelines and best practices for RNA-Seq (Encode Consortium, 2016). Alignment of adapter-trimmed^30^ (Skewer v0.1.123) 2×150 (paired-end; PE) bp strand-specific Illumina reads to the *Mus musculus Mus musculus* GRCm39 mouse genome (assembly accession NCBI: GCA_000001635.9) was achieved with the Spliced Transcripts Alignment to a Reference (STAR v2.5.3a) software^31^, a splice-junction aware aligner. Expression estimation was performed with RSEM^32^ (RNASeq by Expectation Maximization, v1.3.0), generating overall RSEM gene counts. Counts were normalized by TPM. To test for differential gene expression among individual group contrasts, expected read counts obtained from RSEM were used as input into DESeq2^33^ (Version 1. 36.0). Statistical significance of Differentially Expressed genes (DEGs) was defined by a log2fold change of $\pm$(0.5), with statistical significance of the negative-binomial regression test adjusted with a Benjamini-Hochberg FDR correction at the 10% level^34^ and independent filtering requiring genes to have a minimum read count (10 reads) in each group. Heatmap was generated using pheatmap [V.1.0.12] from K-means clustering of all DEGs in the 4-way comparison, with cell value is TPM row-normalized. DOT plots for GO analysis were generated with enrichGO in Clusterprofiler using p-value < 0.05. These raw and processed data sets have been deposited in NCBI’s Gene Expression Omnibus and are accessible through GEO accession number GSE221539. Groups for RNA-seq were sex matched and independently analyzed, with no sex-specific differences identified.

### Statistical analysis

Graphs were generated using Prism 9 (GraphPad Software). Statistical analysis between two groups was run using a student’s unpaired t-test. Multiple groups were compared using ordinary one-way ANOVA with Tukey post hoc test to determine significant comparisons. Statistical significance described as a *p* < 0.05. *P*-values shown as * (*P*$\,\le$0.05). **(*P* $\le$0.01), ***(*P* $\le$0.001), **** (*P* $\le$0.0001). n.s. indicates not significant. Error bars presented as S.E.M.

## Supplementary information

Supplementary Information

## Data Availability

RNA-seq data from WT and *Lrrc10*^*–/–*^ mice are available at the NCBI’s Gene Expression Omnibus (GSE221539). All data are available from the corresponding author upon request.

## References

[1] Murphy SP, Ibrahim NE, Januzzi JL (2020). Heart failure with reduced ejection fraction: a review. JAMA..

[2] Becker RO, Chapin S, Sherry R (1974). Regeneration of the ventricular myocardium in amphibians. Nature.

[3] Cano-Martinez A (2010). Functional and structural regeneration in the axolotl heart (Ambystoma mexicanum) after partial ventricular amputation. Arch. Cardiol. Mex..

[4] Poss KD, Wilson LG, Keating MT (2002). Heart regeneration in zebrafish. Science..

[5] Porrello ER (2011). Transient regenerative potential of the neonatal mouse heart. Science..

[6] Zhu W (2018). Regenerative potential of neonatal porcine hearts. Circulation..

[7] Ye L (2018). Early regenerative capacity in the porcine heart. Circulation.

[8] Sadek H, Olson EN (2020). Toward the goal of human heart regeneration. Cell Stem Cell..

[9] Stockdale WT (2018). Heart regeneration in the mexican cavefish. Cell Rep..

[10] Kobe B, Deisenhofer J (1994). The leucine-rich repeat: a versatile binding motif. Trends Biochem. Sci..

[11] Brody MJ (2012). Ablation of the cardiac-specific gene leucine-rich repeat containing 10 (Lrrc10) results in dilated cardiomyopathy. PLoS One..

[12] Woon MT (2018). Pediatric dilated cardiomyopathy-associated LRRC10 (leucine-rich repeat-containing 10) Variant Reveals LRRC10 as an auxiliary subunit of cardiac L-Type Ca(2+) channels. J. Am. Heart Assoc..

[13] Mahmoud AI, Porrello ER, Kimura W, Olson EN, Sadek HA (2014). Surgical models for cardiac regeneration in neonatal mice. Nat. Protoc..

[14] Adameyko II (2005). Expression and regulation of mouse SERDIN1, a highly conserved cardiac-specific leucine-rich repeat protein. Dev. Dyn..

[15] Kim KH (2007). Lrrc10 is required for early heart development and function in zebrafish. Dev. Biol..

[16] Kim KH, Kim TG, Micales BK, Lyons GE, Lee Y (2007). Dynamic expression patterns of leucine-rich repeat containing protein 10 in the heart. Dev. Dyn..

[17] Porrello ER (2013). Regulation of neonatal and adult mammalian heart regeneration by the miR-15 family. Proc. Natl. Acad. Sci. USA.

[18] Patterson M (2017). Frequency of mononuclear diploid cardiomyocytes underlies natural variation in heart regeneration. Nat. Genet..

[19] Hesse M (2018). Midbody positioning and distance between daughter nuclei enable unequivocal identification of cardiomyocyte cell division in mice. Circ. Res..

[20] Leone M, Magadum A, Engel FB (2015). Cardiomyocyte proliferation in cardiac development and regeneration: a guide to methodologies and interpretations. Am. J. Physiol. Heart Circ. Physiol..

[21] Cui M (2020). Dynamic transcriptional responses to injury of regenerative and non-regenerative cardiomyocytes revealed by single-nucleus RNA sequencing. Dev. Cell.

[22] Quaife-Ryan GA (2017). Multicellular transcriptional analysis of mammalian heart regeneration. Circulation.

[23] O'Meara CC (2015). Transcriptional reversion of cardiac myocyte fate during mammalian cardiac regeneration. Circ. Res..

[24] Houser SR, Piacentino V, Weisser J (2000). Abnormalities of calcium cycling in the hypertrophied and failing heart. J. Mol. Cell Cardiol..

[25] Valdivia CR (2010). Loss-of-function mutation of the SCN3B-encoded sodium channel beta3 subunit associated with a case of idiopathic ventricular fibrillation. Cardiovasc. Res..

[26] Bae J (2021). Malonate promotes adult cardiomyocyte proliferation and heart regeneration. Circulation..

[27] Bersell K, Arab S, Haring B, Kuhn B (2009). Neuregulin1/ErbB4 signaling induces cardiomyocyte proliferation and repair of heart injury. Cell..

[28] Shapiro SD (2014). Cyclin A2 induces cardiac regeneration after myocardial infarction through cytokinesis of adult cardiomyocytes. Sci. Transl. Med..

[29] Manuylov NL, Manuylova E, Avdoshina V, Tevosian S (2008). Serdin1/Lrrc10 is dispensable for mouse development. Genesis..

[30] Jiang H, Lei R, Ding S-W, Zhu S (2014). Skewer: a fast and accurate adapter trimmer for next-generation sequencing paired-end reads. BMC Bioinform..

[31] Dobin A (2013). STAR: ultrafast universal RNA-seq aligner. Bioinformatics..

[32] Li B, Dewey CN (2011). RSEM: accurate transcript quantification from RNA-Seq data with or without a reference genome. BMC Bioinform..

[33] Love MI, Huber W, Anders S (2014). Moderated estimation of fold change and dispersion for RNA-seq data with DESeq2. Genome Biol.

[34] Reiner A, Yekutieli D, Benjamini Y (2003). Identifying differentially expressed genes using false discovery rate controlling procedures. Bioinformatics.

