# Supplementary material for: LRRC10 regulates mammalian cardiomyocyte cell cycle during heart regeneration
Source: NPJ Regen Med. 2023 Jul 28;8:39. doi: 10.1038/s41536-023-00316-0 (PMC10382521; doi:10.1038/s41536-023-00316-0)
Supplement: Supplementary file 1 — Supplementary Information [file 41536_2023_316_MOESM1_ESM.pdf]

Supplementary Information

LRRC10 regulates mammalian cardiomyocyte cell cycle during heart regeneration

Rebecca J. Salamon, Megan C. McKeon, Jiyoung Bae, Xiaoya Zhang, Wyatt G. Paltzer, Kayla N. Wanless, Alyssa R. Schuett, Dakota J. Nuttall, Stephen A. Nemr, Rupa Sridharan, Youngsook Lee, Timothy J. Kamp, Ahmed I. Mahmoud

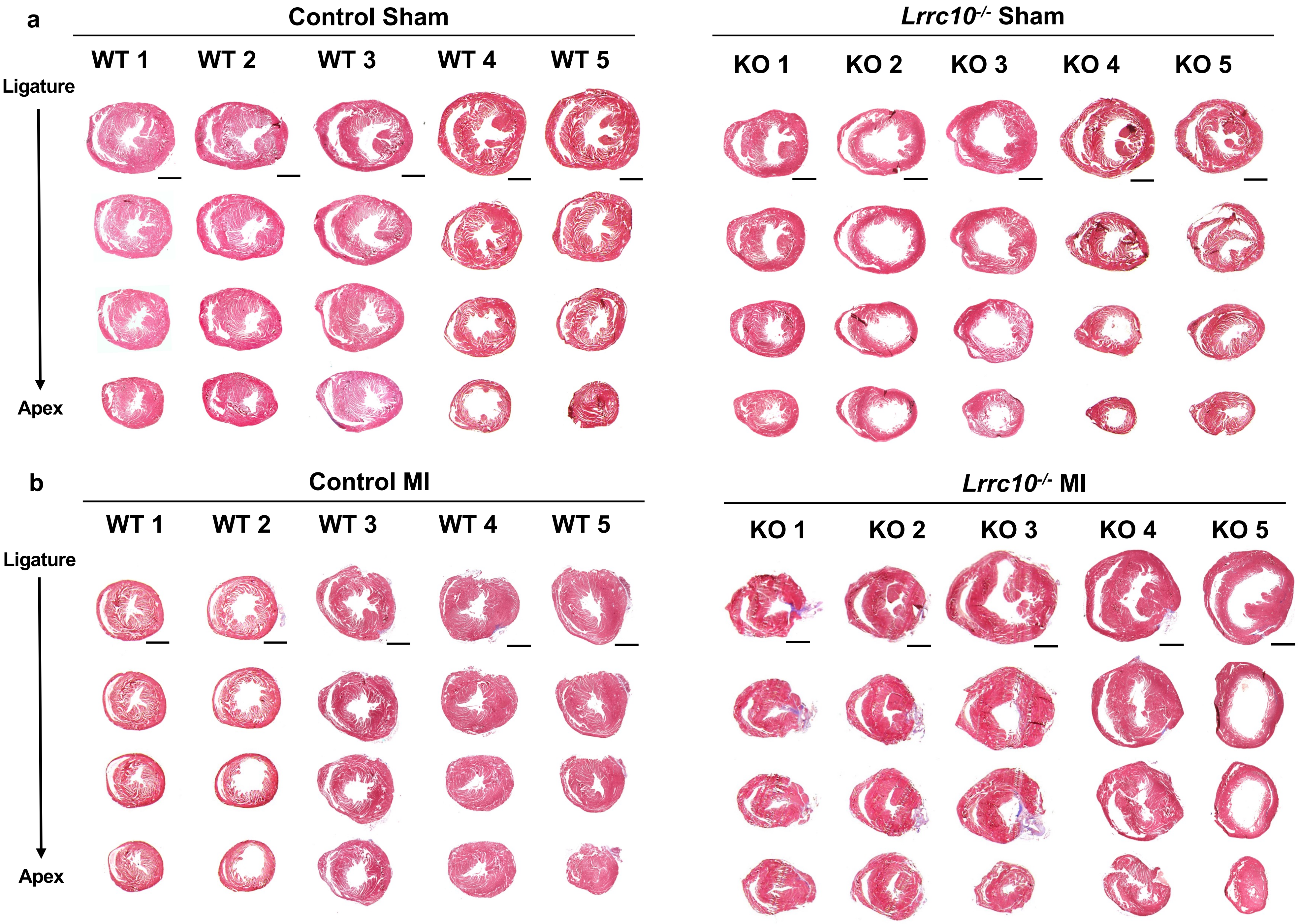

Supplementary Figure 1. ***Lrrc10*$^{-/-}$ hearts show increased scar tissue post-MI**. Masson's Trichrome staining was performed on control (WT) and *Lrrc10*$^{-/-}$ heart sections at 21 days post (a) sham (SH) and (b) MI surgery (n=5); scale bar = 1mm.

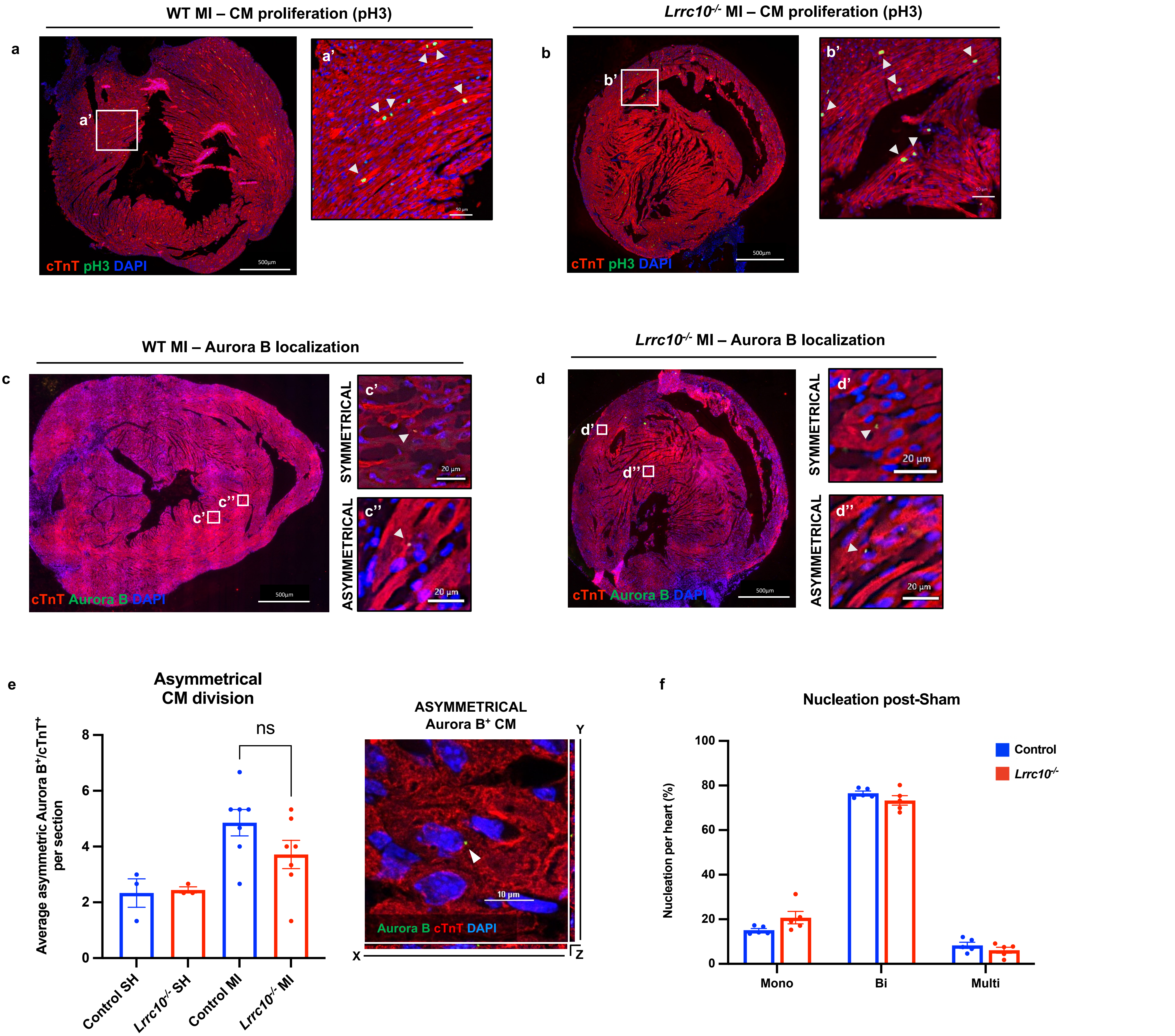

Supplementary Figure 2. ***Lrrc10*$^{-/-}$ hearts show no change in proliferation, asymmetrical cell division, or sham nucleation counts**. At 7 days post-sham or post-MI surgery, there was no difference in cardiomyocyte (CM) proliferation between (a) WT and (b) *Lrrc10*$^{-/-}$ groups, shown as representative sections (scale bar = 500µm) and (a', b') insets (scale bar = 50µm); arrowheads indicate pH3+ CMs. (c) WT and (d) *Lrrc10*$^{-/-}$ hearts show no difference in Aurora B localization, shown as representative sections (scale bar = 500µm) and insets of (c', d') symmetrical (scale bar = 20µm) or (c'', d'') asymmetrical CM division (scale bar = 20µm); arrowhead indicates Aurora B localization between CMs. (e) Post-MI or post-sham surgery, *Lrrc10*$^{-/-}$ hearts show no difference in asymmetrical CM division compared to WT hearts (scale bar = 10µm). (f) Nucleation counts of mono-, bi-, and multinucleated CMs show no difference between WT and *Lrrc10*$^{-/-}$ mice at 14 days post-sham surgery.

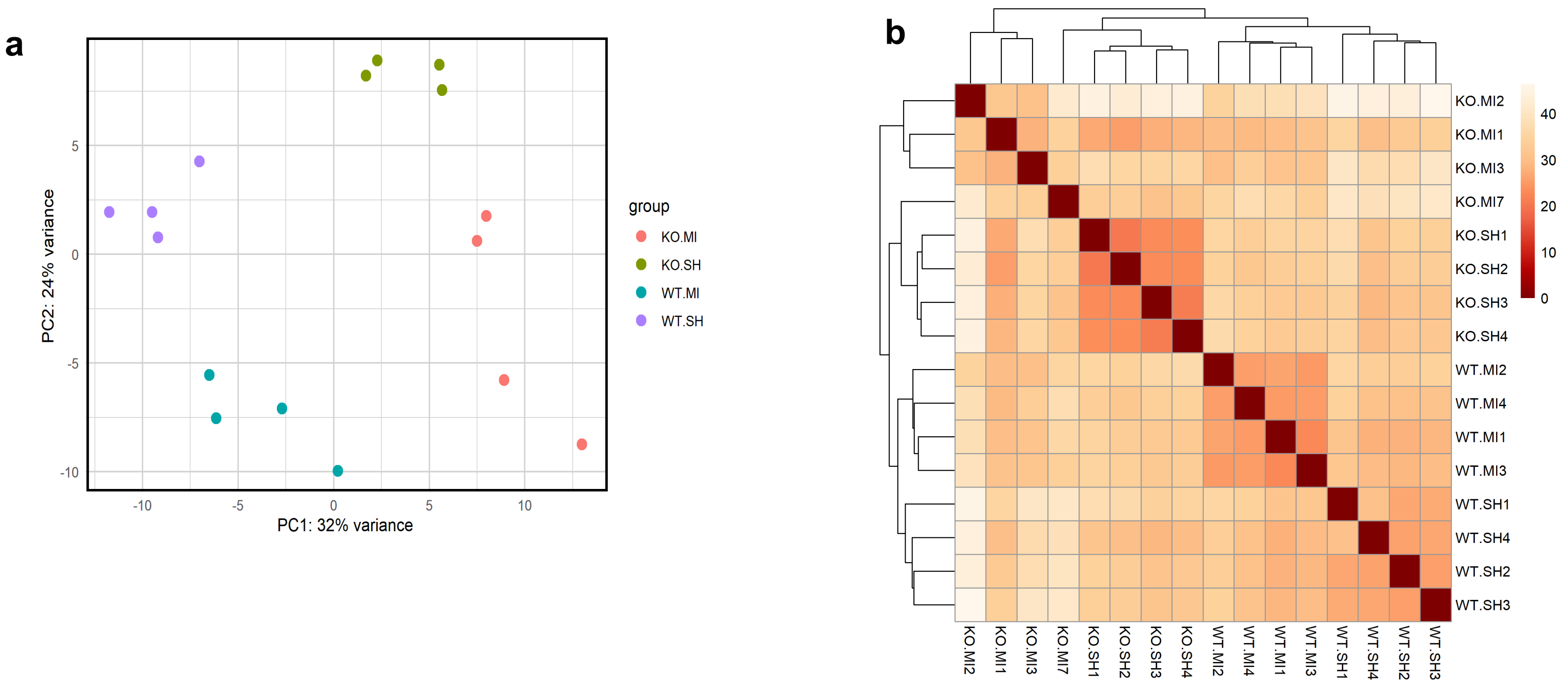

Supplementary Figure 3. Transcriptomic analysis shows unique signature between control and *Lrrc10*$^{-/-}$ hearts post-MI. Bulk RNA sequencing of control and *Lrrc10*$^{-/-}$ mice that underwent a sham (SH) or MI surgery at P1. (a) Principal Component Analysis (PCA) shows separation between different groups. (b) Pearson correlation shows clustering of transcriptomic profiles.

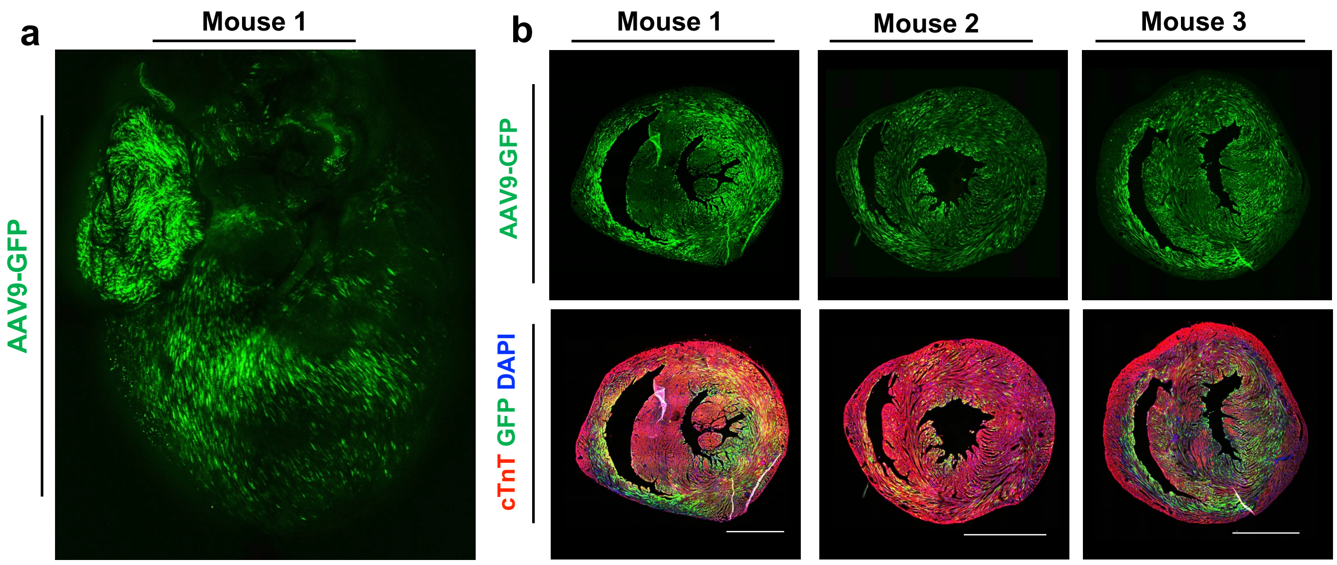

Supplementary Figure 4. Viral control shows successful reporter expression in the postnatal heart. AAV9-cTnT-GFP control vector was injected into P0 mice and hearts were collected at 7 days post-injections. Images were captured of the (a) whole-mount and (b) sectioned hearts (n=3). Scale bar = 1mm.

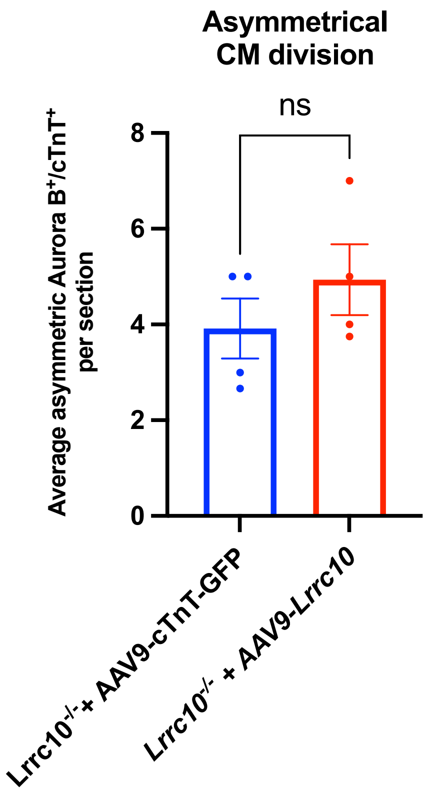

Supplementary Figure 5. Asymmetrical cytokinesis is unchanged between *Lrrc10*$^{-/-}$ mice treated with AAV9-cTnT-GFP or AAV9-cTnT-LRRC10 rescue vector. AAV9-cTnT-GFP (control) or AAV9-cTnT-LRRC10 (rescue) vector was injected into P0 mice. MI surgery was performed at P1, and asymmetrical cytokinesis was measured by Aurora B at 7 days post-MI. No difference in asymmetric cardiomyocyte (CM) division was identified between *Lrrc10*$^{-/-}$ hearts treated with control or rescue vector.

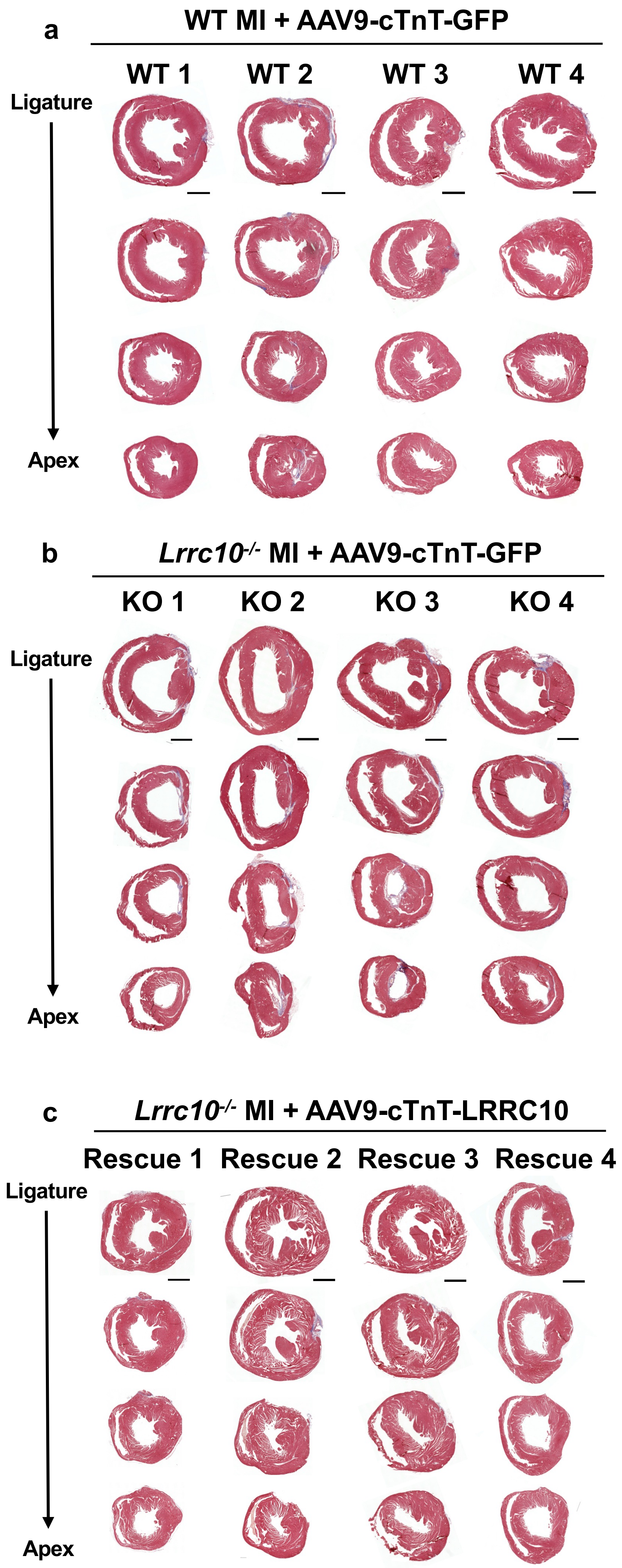

Supplementary Figure 6. ***Lrrc10**$^{-/-}$* **mice treated with AAV9-cTnT-LRRC10 rescue vector promotes regeneration post-MI**. Masson's Trichrome staining of heart sections at 21 days post-MI from WT and *Lrrc10*$^{-/-}$ (KO) mice treated at P0 with (a-b) control AAV9-cTnT-GFP or (c) rescue AAV9-cTnT-LRRC10 vector (n=4); scale bar = 1mm
